# Improved Method for Reliable HMW-GS Identification by RP-HPLC and SDS-PAGE in Common Wheat Cultivars

**DOI:** 10.3390/molecules22071055

**Published:** 2017-06-24

**Authors:** You-Ran Jang, Hye-Rang Beom, Susan B. Altenbach, Min-Ki Lee, Sun-Hyung Lim, Jong-Yeol Lee

**Affiliations:** 1 National Institute of Agricultural Science, RDA, Jeonju 54874, Korea; jang6122@gmail.com (Y.-R.J.); gpfkdcjstk12@naver.com (H.-R.B.); mklee2688@jbnu.ac.kr (M.-K.L.); limsh2@korea.kr (S.-H.L.); 2 USDA-ARS, Western Regional Research Center, 800 Buchanan Street, Albany, CA 94710, USA; Susan.Altenbach@ARS.USDA.GOV

**Keywords:** allelic analysis, high-molecular-weight glutenin subunit, RP-HPLC, SDS-PAGE

## Abstract

The accurate identification of alleles for high-molecular weight glutenins (HMW-GS) is critical for wheat breeding programs targeting end-use quality. RP-HPLC methods were optimized for separation of HMW-GS, resulting in enhanced resolution of 1By and 1Dx subunits. Statistically significant differences in retention times (RTs) for subunits corresponding to HMW-GS alleles were determined using 16 standard wheat cultivars with known HMW-GS compositions. Subunits that were not identified unambiguously by RP-HPLC were distinguished by SDS-PAGE or inferred from association with linked subunits. The method was used to verify the allelic compositions of 32 Korean wheat cultivars previously determined using SDS-PAGE and to assess the compositions of six new Korean cultivars. Three cultivars contained subunits that were identified incorrectly in the earlier analysis. The improved RP-HPLC method combined with conventional SDS-PAGE provides for accurate, efficient and reliable identification of HMW-GS and will contribute to efforts to improve wheat end-use quality.

## 1. Introduction

In addition to improving yield and disease resistance, one of the primary goals of wheat breeding programs is the development of wheat varieties with good end-use quality. End-use quality is largely determined by the gluten proteins consisting of complex groups of proteins referred to as glutenins and gliadins [1]. The quantity and quality of individual proteins within these groups are key factors in determining the unique elasticity and viscosity of wheat dough [2]. Glutenins are divided according to their molecular weights into high-molecular-weight (MW = 67,000–88,000) and low-molecular-weight (MW = 32,000–35,000) glutenin subunits (HMW-GS and LMW-GS, respectively) [3]. The subunits are linked together through disulphide bonds to form some of the largest polymeric proteins found in nature with MWs up to tens of millions. These polymeric proteins affect the rheological properties of wheat dough [4].

HMW-GS are encoded by the *Glu-A1*, *Glu-B1* and *Glu-D1* loci on the long arms of the group I chromosomes [5]. Each locus contains two tightly-linked paralogous genes encoding two different types of HMW-GS, namely the x- and y-type subunits [6,7]. Most common wheat varieties express only three to five subunits because some genes, particularly those encoding the y subunits at the *Glu-A1* locus, are silent [8]. Thus, HMW-GS are divided into five types of proteins; 1Ax encoded at *Glu-A1*, 1Bx and 1By encoded at *Glu-B1*, and 1Dx and 1Dy encoded at *Glu-D1*. The allelic compositions and expression levels of HMW-GS are related to bread-making properties [2,5,9,10]. For example, the presence of 1Dx5 + 1Dy10 subunits has been associated with superior bread-making quality while 1Dx2 + 1Dy12 has been associated with poor bread-making quality, especially dough strength [5,8]. HMW-GS 1Ax2* and 1Dx17 + 1Dy18 also show positive effects on bread-making quality [1,11]. The high expression levels of some HMW-GS also are important for quality [12]. In particular, wheat with two functional copies of the 1Bx7 subunit (1Bx7^OE^) has good dough strength [10,12,13,14].

Over the past decades, several analytical techniques were developed to assess the allelic variation of HMW-GS in wheat breeding programs including SDS-PAGE, two-dimensional gel electrophoresis (2-DGE), reversed-phased high-performance liquid chromatography (RP-HPLC), high-performance capillary electrophoresis (HPCE) and matrix-assisted laser desorption/ionization time-of-flight mass spectrometry (MALDI-TOF-MS) [15,16,17,18,19,20,21,22]. Of these, SDS-PAGE has been used most frequently. SDS-PAGE is simple and easy to perform, but has relatively poor resolution and reproducibility compared to other methods. HMW-GS with similar mobilities are difficult to differentiate by SDS-PAGE and results can vary among laboratories. Since the 1980s RP-HPLC has been used for allelic analysis [16,17,19,20,21]. In RP-HPLC, subunits are separated on a linear gradient of organic solvent and distinguished on the basis of their hydrophobicities. Compared to SDS-PAGE, RP-HPLC separation is extremely precise, fully automated and highly reproducible for identifying and quantifying individual HMW-GS. However, HMW-GS having similar hydrophobicities cannot be differentiated by this method. In particular, there is considerable overlap among many 1Dx and 1By subunits in recent reports. Analyses of HMW-GS by HPCE are more rapid than RP-HPLC. However, multiple peaks are obtained for some HMW-GS subunits, complicating the interpretations of results [20]. MALDI-TOF-MS provides both good resolution and reproducibility, but the instrumentation costs required for the analyses are much higher than the other methods [23]. Thus, MALDI-TOF-MS is not available to many breeding programs. DNA methods also have been developed for the discrimination of HMW-GS alleles. While polymerase chain reaction (PCR) is an inexpensive and rapid method for allele identification, molecular markers are not available for all HMW-GS alleles, particularly By 8*, Dx2.2 and Dx4 (http://maswheat.ucdavis.edu/protocols/FunctionalMarkers/FM_quality.htm).

Unlike many of the wheat producing areas worldwide, the growing period of wheat in South Korea is short due to the rainy season that begins in early June. Prior to 2000, most Korean breeding programs addressed yield and disease resistance. As more recent breeding efforts focus on end-use quality, it has become essential for breeders to select for HMW-GS alleles associated with good bread-making quality. The objective of this study was to develop RP-HPLC methods with improved resolution that could be used along with SDS-PAGE to verify the HMW-GS compositions of Korean wheat varieties. 

## 2. Results and Discussion

### 2.1. Identification of HMW-GS by both RP-HPLC and SDS-PAGE in Standard Cultivars

**Figure 1:**
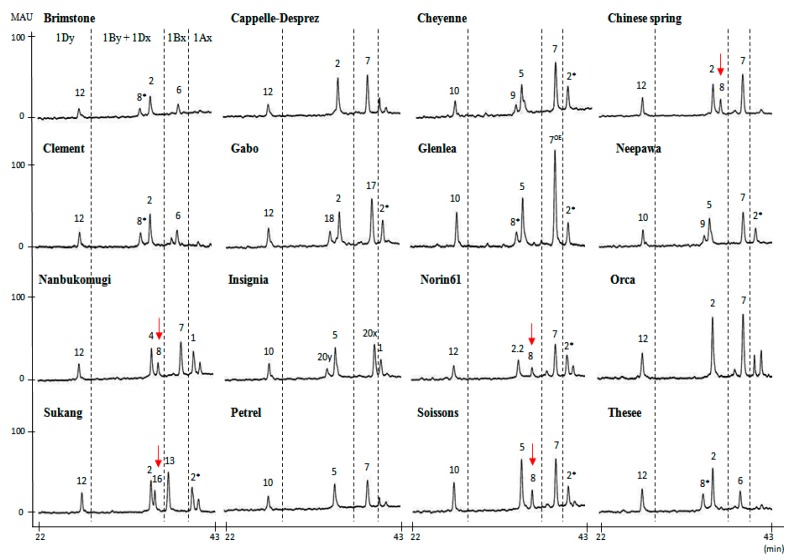
HMW-GS profiles of standard wheat cultivars determined by RP-HPLC. Red arrows point to 1By subunits that elute after 1Dx subunits.

**Figure 2:**
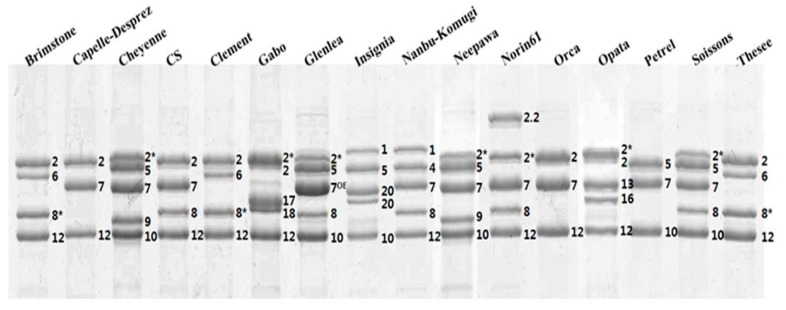
HMW-GS profiles of standard wheat cultivars determined by SDS-PAGE.

**Figure 3:**
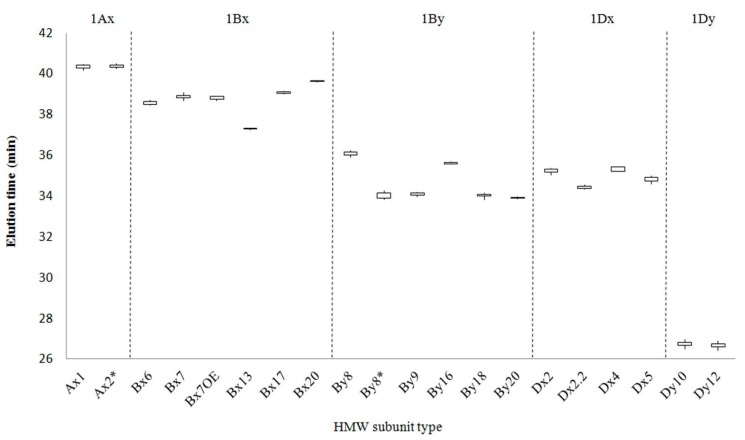
Box and whisker plot of retention times of standard wheat cultivars.

RP-HPLC conditions were optimized for the separation of HMW-GS. A representative chromatogram of reduced and alkylated glutenin from Chinese Spring is shown in Supplementary Figures S1 and S2. The 1Dy12, 1Dx2, 1By8 and 1Bx7 subunits characteristic of this cultivar were well resolved and appear as four distinct peaks between 25 and 42 min. LMW-GS and contaminating gliadins appear as a more complex group of peaks between 42 and 55 min, indicating that HMW-GS are more hydrophilic than LMW-GS. Sixteen standard wheat varieties with the HMW-GS compositions shown in Table 1 were then analyzed by RP-HPLC (Figure 1). The 1Ax, 1Bx, 1By, 1Dx and 1Dy subunits were well resolved by the RP-HPLC separation conditions (Figure 1). In comparison, 1Dx and 1By subunits were not separated in the studies of Dong et al. [19], Gao et al. [20] and Yan et al. [22] although the same RP-HPLC column was used. In this study, a linear gradient of 23–44% ACN for 70 min at 60 °C was used rather than the 21–48% ACN gradient for 55 or 65 min at 50 °C previously reported [19,20,22]. This facilitated the separation of subunits having similar hydrophobicities by increasing the degree of polarity and resulting in extended stays in the column. The 1Dy subunits were the most hydrophilic proteins and were eluted first. In general, these were followed by the 1By, 1Dx, 1Bx and finally the 1Ax subunits. However, the 1By8 subunit in Chinese Spring, Nanbu-komugi, Norin61 and Soissons and the 1By16 subunit in Sukang did not follow this order and eluted after the 1Dx subunits. Retention times (RTs) were determined for each peak in the HMW-GS region and the average RT for each subunit from multiple RP-HPLC runs is reported in Table 2. Relative standard deviations (RSD) less than 0.38% were obtained for all peaks and indicated that the RTs for each subunit were highly reproducible within the experiment. Figure 3 compares the average RTs and the margins of error for each subunit and highlights those HMW-GS that can be readily distinguished on the basis of RT. In particular, subunits that were resolved poorly under the conditions of Dong et al. [19] (1Dx5 and 1By18, 1Dx2 and 1By8, and 1Dx5 and 1By16) were easily separated in the current study (Figure 3). HMW-GS from the standard cultivars also were analyzed by SDS-PAGE (Figure 2).

At the *Glu-A1* locus, 1Ax1, 1Ax2* and null subunits have been described. Standard cultivars Insignia and Nanbu-komugi contain the 1Ax1 subunit with a RT of 40.307 min. Cheyenne, Gabo, Glenlea, Neepawa, Norin61, Sukang and Soissons contain the 1Ax2* subunit characterized by a RT of 40.354 min (Figure 1, Table 1 and Table 2). Because the difference in RT was only 0.05 min, it was not possible to distinguish these two subunits by RP-HPLC. However, the two proteins are readily distinguished on the basis of MW by SDS-PAGE (Figure 2). Standard cultivars Brimstone, Cappelle-Desprez, Chinese Spring, Clement, Orca, Petrel and Thesee contain the null allele and are absent of peaks in this region of the chromatogram (Figure 1).

At the *Glu-B1* locus, most 1Bx and 1By subunits are present in pairs with the exception of 1Bx7 that sometimes occurs alone (Table 1). Six subunits of 1Bx have been described in the standard cultivars; 1Bx6, 1Bx7, 1Bx7^OE^, 1Bx13, 1Bx17 and 1Bx20. Retention times ranged from 37.285 for 1Bx13 in Sukang to 39.603 for 1Bx20 in Insignia (Table 1 and Table 2). Of the 1Bx subunits, 1Bx6, 1Bx7, 1Bx13, 1Bx17 and 1Bx20 were readily distinguished by RTs (Table 2, Figure 3). Subunits 1Bx7 in standard cultivars Cappelle-Desprez, Cheyenne, Chinese Spring, Nanbu-komugi, Neepwa, Norin61, Orca, Petrel and Soissons and 1Bx7^OE^ in Glenlea had similar RTs but could be distinguished by RP-HPLC on the basis of peak height since 1Bx7^OE^ is overexpressed (Figure 1 and Figure 3). 1Bx7 and 1Bx7^OE^ also had similar mobilities in SDS-PAGE. However, 1Bx7 and 1Bx7^OE^ could be distinguished from each other by their relative band intensities and differed from other 1Bx subunits in mobility (Figure 2). Six 1By subunits are represented in the standard cultivars; 1By8, 1By8*, 1By9, 1By16, 1By18 and 1By20. Retention times ranged from 33.894 for 1By20 in the cultivar Insignia to 36.060 for 1By8 in Chinese Spring, Nanbu-komugi, Norin61 and Soissons. Only 1By8 and 1By16 were readily identified by RTs, while 1By9, 1By18 and 1By20 could be distinguished by SDS-PAGE. Both RP-HPLC and SDS-PAGE were required to identify 1By8*; RP-HPLC was required to distinguish 1By8* from 1By8 and 1By16 (Figure 1 and Figure 3) while SDS-PAGE was required to distinguish 1By8* from 1By9, 1By18 and 1By20 (Figure 2).

The 1Dx and 1Dy subunits encoded at the *Glu-D1* locus are present in pairs. Four 1Dx HMW-GS have been described, 1Dx2, 1Dx2.2, 1Dx4 and 1Dx5. 1Dx2.2 with a RT of 34.428 and 1Dx5 with an RT of 34.792 were easily identified and both could be distinguished from 1Dx2 and 1Dx4 with RTs of 35.195 and 35.269. But, it was not possible to distinguish 1Dx2 and 1Dx4 by RP-HPLC (Figure 1 and Figure 3; Table 2). SDS-PAGE was required to distinguish the 1Dx2 subunit in Brimstone, Cappelle-Desprez, Chinese Spring, Clement, Gabo, Orca, Sukang and Thesee from the 1Dx4 subunit in Nanbu-komugi (Figure 2). Two 1Dy subunits have been described, 1Dy10 and 1Dy12. While these proteins are well separated from other HMW-GS by RP-HPLC, the two subunits cannot be distinguished because of their very similar RTs (Figure 3, Table 2). 1Dy10 and 1Dy12 also have very similar mobilities in SDS-PAGE (Figure 2). However, the 1Dy10 subunit always pairs with the 1Dx5 subunit while the 1Dy12 subunit occurs with 1Dx2, 1Dx2.2 or 1Dx4. As a result, the identities of the 1Dy subunits could be inferred once the identities of the 1Dx subunits were determined.

Since 1By8 and 1By16 elute later than other 1By and 1Dx subunits in RP-HPLC, the identities of these subunits were confirmed. Fractions corresponding to the four HMW-GS RP-HPLC peaks in the standard cultivar Chinese Spring and the five peaks in Opata were collected manually, dried and reanalyzed by SDS-PAGE (Supplementary Figure S2). This analysis confirmed that the order of elution was 1Dy12, 1Dx2, 1By8 and 1Bx7 for Chinese Spring and 1Dy12, 1Dx2, 1By16, 1Bx13 and 1Ax2* for Opata. The data suggest that the surface hydrophobicities of the 1By8 and 1By16 subunits are higher than other 1By and 1Dx subunits.

As can be seen from the above results, RP-HPLC alone could not be used to predict allelic compositions of HMW-GS because some subunits show little difference in RT (Figure 3). Similarly, SDS-PAGE alone could not be used for this purpose because some subunits have very similar mobilities (Figure 2). However, by combining the two methods it was possible to obtain accurate assessments of the HMW-GS compositions of wheat cultivars.

### 2.2. Compositional Analysis of HMW-GS in 38 Korean Wheat Cultivars

**Figure 4:**
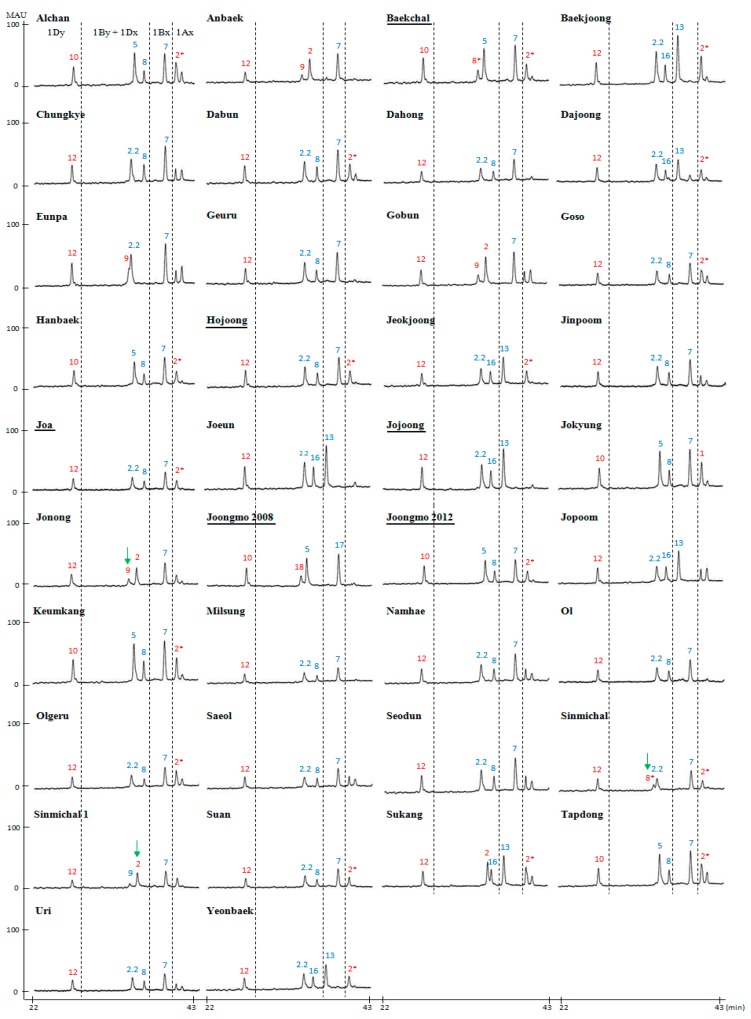
HMW-GS profiles of 38 Korean wheat cultivars determined by RP-HPLC. Subunits that could not be distinguished unequivocally by RT are indicated in red. Their identities were confirmed by SDS-PAGE. Arrows denote subunits that were incorrectly identified in previous SDS-PAGE analyses. Names of new cultivars that were not analyzed previously by SDS-PAGE are underlined.

**Figure 5:**
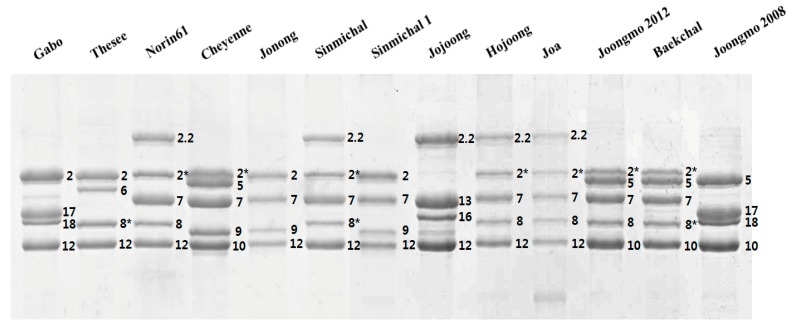
HMW-GS profiles of Korean wheat cultivars determined by SDS-PAGE. HMW-GS in Jonong, Sinmichal and Sinmichal 1 were incorrectly identified in previous SDS-PAGE analyses. HMW-GS from Jojoong, Hojoong, Joa, Joongmo 2012, Baekchal and Joongmo 2008 were determined in this study. Profiles of standard cultivars Gabo, Thesee, Norin61 and Cheyenne are shown for comparison.

To determine the feasibility of the combined methods for use in breeding programs, the improved RP-HPLC method was used to assess the HMW-GS compositions of 38 Korean wheat cultivars (Figure 4, Table 3). Thirty-two cultivars had been analyzed previously by SDS-PAGE [28,29]. With the exception of the cultivars Jonong, Sinmichal and Sinmichal 1, the assignments of the 32 cultivars determined by RP-HPLC confirmed those obtained previously by SDS-PAGE. The 1By subunit was identified incorrectly in both Jonong and Sinmichal while the 1Dx subunit was identified incorrectly in Sinmichal 1. Both Jonong and Sinmichal contained peaks with RTs of 33.942 and 34.091, respectively, indicating that the 1By subunit was either 1By8*, 1By9, 1By18 or 1By20 rather than 1By8 with an RT of 36.060. Reanalysis of Jonong and Sinmichal by SDS-PAGE (Figure 5) revealed that Jonong contains 1By9 while Sinmichal contains 1By8*. In Sinmichal 1, a peak was identified with an RT of 35.195, indicating that the 1Dx subunit was 1Dx2 rather than 1Dx2.2 with an RT of 34.428. Again, reanalysis of the proteins by SDS-PAGE demonstrated that the subunit was clearly 1Dx2 (Figure 5). 

The compositions of six new cultivars (Baekchal, Hojoong, Jojoong, Joa, Joongmo 2008, Joongmo 2012) were then determined using both RP-HPLC and SDS-PAGE (Figure 4 and Figure 5). Of the new cultivars, the HMW-GS composition of Jojoong was determined using only RP-HPLC (Figure 4, Table 3). For Hojoong, Joa and Joongmo 2012, the identities of all HMW-GS except the 1Ax subunits were distinguished easily by RP-HPLC (Figure 4, Table 3). SDS-PAGE was required to identify the 1Ax2* subunits in these cultivars (Figure 5). For Joongmo 2008, the identities of all HMW-GS except the 1By subunits were determined by RP-HPLC (Figure 4, Table 3). This cultivar was found to contain 1By18 by SDS-PAGE (Figure 4). Both RP-HPLC and SDS-PAGE also were required for accurate assignment of HMW-GS composition in Baekchal (Figure 4 and Figure 5, Table 3). The above analysis demonstrates that the improved RP-HPLC method combined with conventional SDS-PAGE can be used for accurate, efficient and reliable identification of HMW-GS alleles. As a result, the method will contribute to breeding efforts to improve wheat end-use quality.

## 3. Experimental Section

### 3.1. Plant Materials

Grain from standard wheat (*Triticum aestivum* L.) cultivars with known HMW-GS compositions was kindly provided by National Plant Germplasm System of the USDA-ARS, Albany, CA, USA. Grain from 38 Korean hexaploid wheat cultivars was harvested in 2014 and 2015 by RDA National Institute of Crop Sciences, Jeonju, Korea. Grain was crushed with a cyclone sample mill (Udy Corporation, Fort Collins, CO, USA). 

### 3.2. Glutenin Extraction

The glutenin was extracted using the procedure of Singh et al. [30]. One hundred mg of flour was extracted with 5 mL of 50% (*v*/*v*) propanol for 30 min at 65 °C. After centrifugation at 10,000× *g* for 5 min, the supernatant fraction containing gliadin was removed. This extraction was repeated on the pellet two additional times. The precipitate was then extracted with 0.5 mL 50% (*v*/*v*) propanol, 0.08 M Tris-HCl pH 8.0 containing 1% (*w*/*v*) dithiothreitol (DTT) at 65 °C for 30 min. After centrifugation at 10,000× *g* for 5 min, 0.5 mL of the same buffer containing 1.4% 4-vinylpyridine (*v*/*v*) instead of 1% DTT was added for alkylation at 65 °C for 15 min. After centrifugation at 10,000× *g* for 2 min, the supernatant was transferred to a new 1.5 mL tube and stored at −20 °C overnight. For RP-HPLC and SDS-PAGE the extracted glutenin fractions were precipitated using 15% (*v*/*v*) TCA/acetone at −20 °C.

### 3.3. Analysis of the Individual HMW-GS Using RP-HPLC

The analysis of HMW-GS was modified from Gao et al. [20]. HMW-GS were analyzed by RP-HPLC using a Waters Alliance e2695 equipped with Agilent ZORBAX 300SB-C_18_ column (5 µm, 4.6 × 250 mm i.d., Agilent Technologies, Santa Clara, CA, USA). The solvents (A) water and (B) acetonitrile (ACN), both containing 0.1% (*v*/*v*) trifluoroacetic acid were used as the mobile phase. Glutenin pellets were mixed completely in 500 µL of 0.1% TFA in 20% ACN and filtered using a PVDF syringe filter (0.45 µm, Whatman, Maidstone, UK). Ten µL of each sample was injected. Proteins were eluted with a linear gradient of 23–44% of 0.1% TFA in 20% ACN over 70 min. The RP-HPLC analysis of HMW-GS was carried out with a flow rate of 0.8 mL/min at a column oven temperature of 60 °C and monitored at a wavelength of 206 nm. Individual peaks of HMW-GS for Chinese Spring and Opata were manually collected in 15 mL Falcon-tubes, and dried by centrifugal vacuum evaporator (HT-4X, GeneVac Ltd., Ipswich, UK) for 4 h. Each fraction was dissolved in 1 mL of 50% propanol and analyzed by SDS-PAGE.

For statistical analyses, retention times for each subunit were averaged from the replicate RP-HPLC analyses of the standard cultivars. Standard deviations and relative standard deviations (RSD %) were determined for each HMW-GS subunit and a box and whiskers plot of the dataset was constructed using Excel.

### 3.4. SDS-PAGE

SDS-PAGE was carried out using the method described by Lee et al. [31] and performed on Hoefer SE 250 Mighty Small II electrophoresis units. The resulting glutenin pellets and 70 µL of sample buffer (50 mM Tris-HCl pH 6.8, 2% β-mercaptoethanol, 2% SDS, 20% glycerol and 0.01% bromophenol blue) were mixed completely. Three µL of each sample was visualized on 12.5% SDS-PAGE gels stained with Coomassie Brilliant Blue R-250.

## Figures and Tables

**Table 1:** Allelic compositions of HMW-GS in standard cultivars.

Cultivar	HMW-GS			Reference
	*Glu-A1*	*Glu-B1*	*Glu-D1*	
Brimstone	N	6 + 8*	2 + 12	Liu et al. [24]
Cappelle-Desprez	N	7	2 + 12	Liu et al. [24]
Cheyenne	2*	7 + 9	5 + 10	Dupont et al. [25]
Chinese Spring	N	7 + 8	2 + 12	Liu et al. [24]
Clement	N	6 + 8*	2 + 12	Liu et al. [24]
Gabo	2*	17 + 18	2 + 12	Liu et al. [24]
Glenlea	2*	7^OE^ + 8*	5 + 10	Naeem and Sapirstein [21]
Insignia	1	20 + 20	5 + 10	Branlard [26]
Nanbu-komugi	1	7 + 8	4 + 12	Liu et al. [24]
Neepwa	2*	7 + 9	5 + 10	Liu et al. [24]
Norin61	2 *	7 + 8	2.2 + 12	Hua et al. [27]
Orca	N	7	2 + 12	Liu et al. [24]
Sukang ^a^	2*	13 + 16	2 + 12	Park et al. [28]
Petrel	N	7	5 + 10	Liu et al. [24]
Soissons	2*	7 + 8	5 + 10	Liu et al. [24]
Thesee	N	6 + 8*	2 + 12	Liu et al. [24]

^a^ Cultivar was used in place of standard cultivar Opata and has identical HMW-GS.

**Table 2:** Reproducibility of RP-HPLC retention times for HMW-GS in standard wheat cultivars.

Type	HMW-GS	Number of Cultivars	Total Number of Analyses	Average Retention Time (min)	RSD (%)
Ax	Null	7			
	1	2	9	40.307 ± 0.078	0.195
	2*	7	18	40.354 ± 0.077	0.190
Bx	6	3	8	38.516 ± 0.059	0.154
	7	9	25	38.842 ± 0.077	0.199
	7^OE^	1	6	38.770 ± 0.078	0.201
	13	1	3	37.285 ± 0.030	0.081
	17	1	5	39.075 ± 0.053	0.138
	20x	1	4	39.603 ± 0.061	0.155
By	8	4	12	36.060 ± 0.083	0.231
	8*	4	10	33.948 ± 0.098	0.290
	9	2	5	34.091 ± 0.085	0.252
	16	1	3	35.604 ± 0.039	0.109
	18	1	5	34.022 ± 0.108	0.319
	20y	1	3	33.894 ± 0.043	0.127
Dx	2	8	24	35.195 ± 0.114	0.325
	2.2	1	3	34.428 ± 0.082	0.238
	4	1	3	35.269 ± 0.094	0.267
	5	6	17	34.792 ± 0.119	0.343
Dy	10	6	10	26.736 ± 0.101	0.377
	12	10	18	26.528 ± 0.098	0.370

**Table 3:** Allelic compositions of HMW-GS in Korean cultivars by RP-HPLC. Most cultivars were analyzed previously by SDS-PAGE. Subunits that differed in the two analyses are indicated in bold.

Cultivar	Pedigree	HMW-GS		
		*Glu-A1*	*Glu-B1*	*Glu-D1*
Alchan	Suwon210/Tapdong	2*	7 + 8	5 + 10
Anbaek	Sae/Geuru	N	7 + 9	2 + 12
Baekchal ^a^	Sinmichal /Keumkang	2*	7 + 8*	5 + 10
Baekjoong	Keumkang/Olgeuru	2*	13 + 16	2.2 + 12
Chungkye	Norin4/Sharbatisonora	N	7 + 8	2.2 + 12
Dabun	Suwon234//sw76039/Suwon220/3/Keumkang	2*	7 + 8	2.2 + 12
Dahong	Norin72/Wonkwang	N	7 + 8	2.2 + 12
Dajoong	SW992114-NM-131-7/Gobun	2*	13 + 16	2.2 + 12
Eunpa	Chugoku81/3/Tob-CNO//Yuksung3/Suwon185	N	7 + 9	2.2 + 12
Geuru	Strampelli/69D-3607//Chokwang	N	7 + 8	2.2 + 12
Gobun	Eunpa/Tapdong//Eunpa/Shannung6521	N	7 + 9	2 + 12
Goso	Gobun/Ol	2*	7 + 8	2.2 + 12
Hanbaek	Shann7859/Keumkang//Guamuehill	2*	7 + 8	5 + 10
Hojoong ^a^	Alchan *2/3/Chunm18//JUP/BJY/4/Keumkang	2*	7 + 8	2.2 + 12
Jeokjoong	Keumkang/Tapdong	2*	13 + 16	2.2 + 12
Jinpoom	Geuru/Genaro81	N	7 + 8	2.2 + 12
Joa ^a^	SW86054-MB-27-3-2-1-1-1/Sumai#3	2*	7 + 8	2.2 + 12
Joeun	Eunpa/Suwon242	N	13 + 16	2.2 + 12
Jojoong ^a^	Suwon272/Olgeuru//Keumkang/Suwon252	N	13 + 16	2.2 + 12
Jokyung	Seri82/Keumkang	1	7 + 8	5 + 10
Jonong	Suwon234/SW80199	N	7 + 9	2 + 12
Joongmo2008 ^a^	Eunpa *2//SH3/CBRD/3/Keumkang	N	17 + 18	5 + 10
Joongmo2012 ^a^	Sinmichal/Keumkang	2*	7 + 8	5 + 10
Jopoom	Kanto75//OR8500494P/Bezostaya	N	13 + 16	2.2 + 12
Keumkang	Geuru/Kanto75//Eunpa	2*	7 + 8	5 + 10
Milsung	Shirogane//Norin43/Sonalika	N	7 + 8	2.2 + 12
Namhae	Ol/Calidad	N	7 + 8	2.2 + 12
Ol	Norin72/Norin12	N	7 + 8	2.2 + 12
Olgeuru	Geuru/Chokwang//Saikai143	2*	7 + 8	2.2 + 12
Saeol	Shirogane//Norin43/Sonalika	N	7 + 8	2.2 + 12
Seodun	Geuru/Genaro81	N	7 + 8	2.2 + 12
Sinmichal	Olgeuru//Kanto107/BaiHuo	2*	7 + 8*	2.2 + 12
Sinmichal1	Alchan//Kanto107/BaiHuo	N	7 + 9	2 + 12
Suan	Keumkang/Eunpa//Keumkang	2*	7 + 8	2.2 + 12
Sukang	Suwon266/Asakaze	2*	13 + 16	2 + 12
Tapdong	Chugoku81//Suwon158/Toropi	2*	7 + 8	5 + 10
Uri	Geuru/Ol	N	7 + 8	2.2 + 12
Younbaek	Keumkang/Tapdong	2*	13 + 16	2.2 + 12

^a^ new Korean wheat cultivars.

## Supplementary Materials

The supplementary materials are available online.
