# Supplementary figures and images for: Improved Method for Reliable HMW-GS Identification by RP-HPLC and SDS-PAGE in Common Wheat Cultivars
Source: Molecules. 2017 Jun 24;22(7):1055. doi: 10.3390/molecules22071055 (PMC6152065; doi:10.3390/molecules22071055)

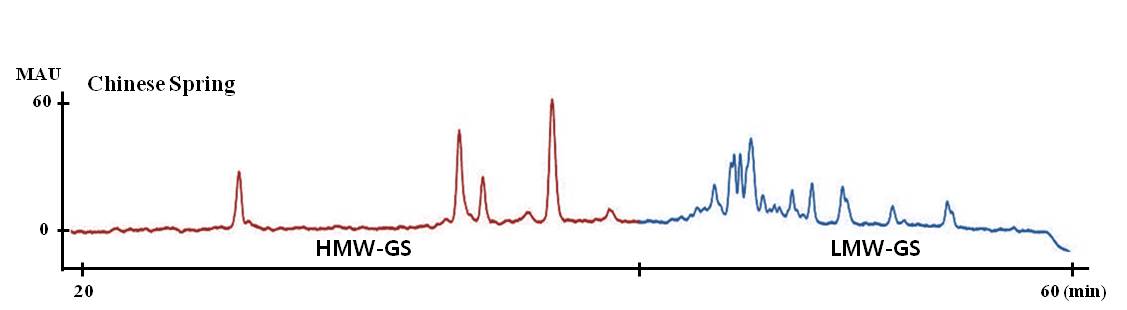

Supplement: Supplementary file 1 [file molecules-22-01055-s001.zip › supplementary files/Supplementary Figure S1.jpg]

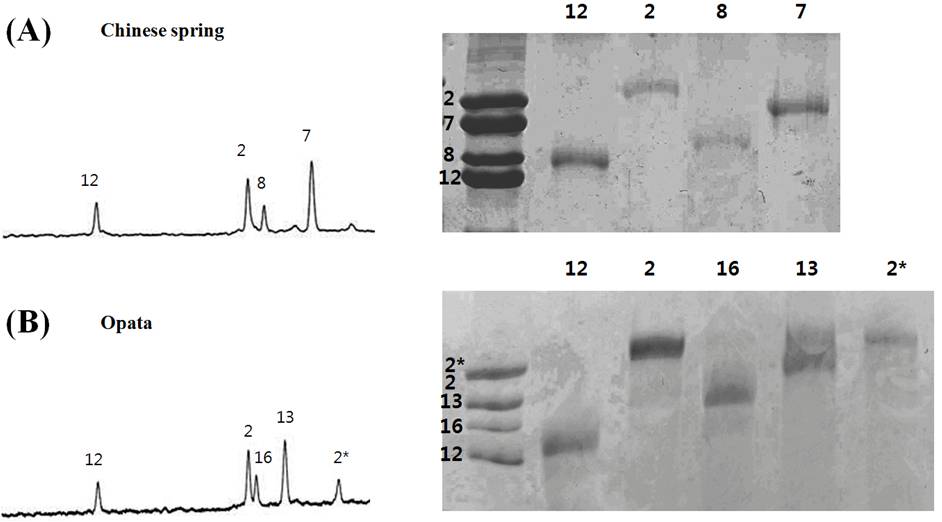

Supplement: Supplementary file 1 [file molecules-22-01055-s001.zip › supplementary files/Supplementary Figure S2.jpg]
